# Relinquishing Anonymity in Living Donor Kidney Transplantation: Lessons Learned From the UK Policy for Anonymous Donors

**DOI:** 10.3389/ti.2022.10091

**Published:** 2022-02-04

**Authors:** Mathilde C. Pronk, Lisa Burnapp, Marlies E. J. Reinders, Emma K. Massey

**Affiliations:** ^1^ Erasmus MC Transplant Institute, University Medical Center Rotterdam, Rotterdam, Netherlands; ^2^ Department of Transplantation and Nephrology, Guy’s & St Thomas’ NHS Foundation Trust, London, United Kingdom

**Keywords:** kidney transplantation, living donation, Medical Ethics, kidney exchange, anonymity, non-directed altruistic donation

## Abstract

Anonymous living donor kidney transplantation (LDKT) is performed in many countries and policies on anonymity differ. The UK is the only European country with a conditional policy, allowing pairs to break anonymity post-transplant. There is little evidence on how contact after anonymous LDKT is experienced. In this cross-sectional study participants who donated or received a kidney through non-directed altruistic kidney donation or within the UK living kidney sharing scheme completed a questionnaire on their experiences with and attitudes towards anonymity. Non-parametric statistics were used to analyse the data. 207 recipients and 354 donors participated. Anonymity was relinquished among 11% of recipients and 8% of donors. Non-anonymous participants were generally content with non-anonymity. They reported positive experiences with contact/meeting the other party. Participants who remained anonymous were content with anonymity, however, 38% would have liked to meet post-transplant. If the other party would like to meet, this number increased to 64%. Although participants agreed with anonymity before surgery, they believe that, if desired, a meeting should be allowed after surgery. UK donors and recipients were satisfied with conditional anonymity and experiences with breaking anonymity were positive. These results support the expansion of conditional anonymity to other countries that allow anonymous LDKT.

## Graphical Abstract

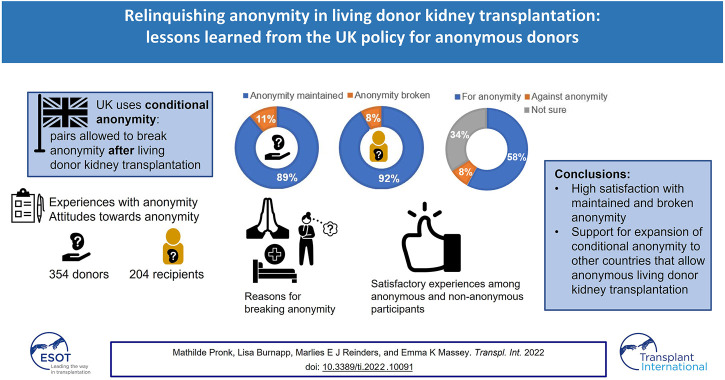

## Introduction

Living donor kidney transplantation (LDKT) is the treatment of choice for patients with end-stage kidney disease. Changes to national legal frameworks and policies have enabled the growth of living donor programmes through both innovative approaches in clinical practice [e.g., kidney exchange programmes (KEPs) and antibody incompatible transplantation] and expansion of the donor pool—from genetically related donors, to inclusion of emotionally related donors (spouses, friends), and even strangers. Donation of a kidney from a living person to a stranger (without knowing the identity or any characteristics of the recipient before transplantation) is known as non-directed altruistic donation (NDAD), but it is also described as unspecified kidney donation (UKD), anonymous or “Good Samaritan” donation (1). Non-directed altruistic donors (NDADs) often donate into KEPs to initiate chains of transplants that complete with a recipient on the national transplant list. This is allowed in many countries, such as Australia, Canada, Netherlands, United Kingdom and United States (2). Within Europe, KEPs are especially well established in the Netherlands and in the United Kingdom (3). In these countries, transplants from NDADs make an invaluable contribution to the living donor pool, currently accounting for around 8% (36 NDADs) in the Netherlands (4) to 10% (100 NDADs) in the UK (5) allowing a greater number of transplants to be carried out in these schemes. A detailed description of KEPs in Europe has been provided by Biro et al. (6).

Anonymity of donors and recipients in KEPs is complex and approaches vary between countries based on national policies (6). Anonymity can be absolute (i.e., applicable before and after surgery without permissible exceptions) or conditional (i.e., allowing removal of anonymity under certain circumstances). The advantages and disadvantages of both approaches are well described in an opinion paper by Mamode et al. (7) in which it was concluded that there is compelling evidence for maintaining anonymity of both parties before and after transplantation. However, requiring absolute anonymity when donors and recipients wish to break it, has been perceived as paternalistic by both transplant professionals (7) and donors and recipients who had participated in KEPs in the Netherlands and Sweden (8, 9). In these studies, the experience of anonymity in the Netherlands and Sweden was investigated both retrospectively and prospectively. In general, donors and recipients were satisfied with absolute anonymity, and only a minority of participants would have liked to meet the other party. However, both studies revealed that more than half of all donors and recipients would be open for a meeting if the other party desired that. Moreover, regardless of personal experience or desire for contact, the dominant opinion was that the decision to have contact or meet should be left up to the individuals themselves (8, 9).

These studies were conducted in the Netherlands and Sweden where anonymity is absolute. This differs from the policy in the UK where anonymity is conditional, i.e., anonymity can be revoked by mutual consent of both donor and recipient after surgery. We do not know to what extent donors and recipients in the UK use this option nor their experiences of revoking anonymity. This data is critical in evaluating the effects of maintaining or revoking anonymity after transplantation. The principal aim of this study was to assess the proportion of UK donors and recipients that maintained/broke anonymity after donation or transplantation and to understand their experiences. Secondly, we aimed to assess the attitudes of the UK donors and recipients towards the principle of anonymity and whether these attitudes differed between donors and recipients and between the donors and recipients for whom anonymity was maintained or broken.

## Materials and Methods

### Participants and Procedure

Donors and recipients (≥18 years old) who anonymously donated or received a living donor kidney in the period 2010–2014, with a minimum of 1 year after surgery, were considered for inclusion. These included donor-recipient pairs who participated in paired-pooled donation (part of the UK living kidney sharing scheme, UKLKSS), as well as NDADs and recipients on the UK transplant list. Data collection took place in November and December 2016. 618 Donors and 584 recipients were identified from the electronic UK Transplant Database and invited to participate by a letter from NHS Blood and Transplant. The letter included information on the study and the questionnaire on anonymity. One reminder was sent to non-responders. The participants completed the questionnaire on paper. Informed consent was assumed by completion and return of the questionnaire. The study protocol received UK Research Ethics Committee approval (NHSBT ID: 16NS0002).

### Measures

#### Socio-Demographic and Medical Characteristics

Self-reported socio-demographic and medical characteristics of participants can be found in Table 1.

**TABLE 1:** Socio-demographic and medical characteristics of participants.

	Recipients (n = 204)		Donors (n = 354)		*p* Value
	n	%	n	%	
Age at operation	186		299		0.003
Median (range)	54 (18–76)		58 (21–85)		
Gender	196		349		0.03
Male	78	40	172	50	
Female	118	60	177	50	
Highest education achieved	194		346		n.s.
Secondary school	58	30	97	28	
Further education	136	70	249	72	
Transplant program	196		346		0.008
UK Transplant list/NDAD^1^	98	50	217	63	
Paired pooled recipient/donor	98	50	129	37	
Median months since surgery (range)	188		302		n.s.
	42 (16–93)		40 (23–82)		
Preemptive transplantation	197				
Yes	36	18			
Median months on dialysis before transplantation (range)	156				
	29 (1–240)				
Number of transplants	197				
1	143	73			
2	41	21			
3	13	6			

1 NDAD, non-directed altruistic donor.

#### Experiences With (Revoked) Anonymity and Attitude Towards Anonymity

We used a questionnaire on anonymity, originally developed by a European platform on Ethical, Legal and Psychosocial Aspects of organ Transplantation (ELPAT) and refined by a Dutch research team of transplantation specialists (8). This was adapted for the UK cohort, including adding some country specific questions. In the questionnaire, anonymity was defined as “not knowing from whom the kidney is received or to whom the kidney is donated, except for general characteristics, such as gender or age.” The questionnaire consisted of closed and open-ended items that measured experiences with and attitudes towards (revoked) anonymity. The items that were used to measure experiences with anonymity are displayed in Table 2. General attitude towards anonymity between donors and unknown recipients was measured (for, against, not sure). A further 12 statements were assessed using 1–7 point Likert scales, as shown in Table 3.

**TABLE 2:** List of questions measuring experiences with anonymity.

How stressful did you find the donation/transplantation?	1 = not stressful at all; 7 = very stressful
How content are you with your decision to donate your kidney?	1 = completely discontent; 7 = completely content
What do you know about the official policy on anonymity in the UK?	
Anonymity was required both before and after donation	
Anonymity was required before donation, but after donation donor and recipient can meet if both parties agree	
No official policy on anonymity	
Don’t know	
When anonymity was maintained:	
How content were you with being anonymous to your donor/recipient before donation?	1 = completely discontent; 7 = completely content
How content were you with being anonymous to your donor/recipient after donation?	1 = completely discontent; 7 = completely content
Would you have liked to have had contact with or meet the donor/recipient of your kidney before donation?	Yes/No/Not sure
Would you have liked to have had contact with or meet the donor/recipient of your kidney after donation?	Yes/No/Not sure
If the donor/recipient would like to make contact with you or meet you, would you be open to such contact/meeting?	Yes/No/Not sure
Did you send an anonymous card, letter or similar item to the donor/recipient?	Yes/No
Did you receive an anonymous card, letter or similar item from the donor/recipient?	Yes/No
When anonymity was broken:	
How was anonymity broken? (multiple answers are possible)	
I had contact with the donor/recipient ……………. month(s) after donation (e.g., by social media, writing e-mails or speaking on the phone)	
I met the donor/recipient in person ………………… month(s) after donation	
We accidentally found out about each other (e.g. through (social) media)	
We accidentally met each other	
Who initiated this contact or meeting?	
I initiated contact with the donor/recipient	
A member of my family/friend initiated contact with the donor/recipient	
The donor/recipient initiated contact with me	
A member of the donor’s/recipient’s family/friend initiated contact with me	
Not applicable: we found out about each other or met accidentally	
How content were you with anonymity before donation?	1 = completely discontent; 7 = completely content
How content are you with the fact that your donor/recipient is NOT anonymous to you?	1 = completely discontent; 7 = completely content
How did you experience the contact or meeting with the donor/recipient?	1 = very negatively; 7 = very positively
Do you regret having contact with or meeting the donor/recipient?	1 = not at all; 7 = a great deal

**TABLE 3:** Attitude statements for recipients and donors.

	Recipients *n* = 204			Donors *n* = 354			*p-*Value
Statements^1^	Mdn	IQR	*n*	Mdn	IQR	*n*	
There must be anonymity between donor and recipient BEFORE surgery	7	4–7	199	7	5–7	352	n.s.
There must be anonymity between donor and recipient AFTER surgery	4	2–6	197	4	2–6	352	n.s.
If both parties agree, the donor and recipient should be allowed to meet BEFORE surgery	4	2–7	201	3	1–6	351	n.s.
If both parties agree, the donor and recipient should be allowed to meet AFTER surgery	7	4–7	198	7	5–7	349	n.s.
The donor has the right to remain anonymous	7	7–7	202	7	7–7	350	n.s.
The recipient has the right to remain anonymous	7	7–7	202	7	7–7	349	n.s.
The donor has the right to know to whom he/she is donating a kidney	2	1–5	201	1	1–3	349	0.000
The recipient has the right to know from whom he/she is receiving a kidney	1	1–4	201	1	1–4	348	n.s.
Anonymity makes a donation altruistic	6	4–7	189	6	3–7	337	n.s.
The donation should only proceed if the donor agrees to anonymity	4	1–6	200	4	1–7	340	n.s.
If the donation procedure was not anonymous, more people would donate their kidney altruistically to a stranger	3	1–4	198	3	2–4	340	n.s.
In practice, anonymity is difficult to maintain	2	1–4	200	1	1–3	347	n.s.

1 All statements are scored on a 7-point Likert scale (1 = completely disagree—7 completely agree).

### Statistical Analyses

Descriptive statistics were used to describe the participants’ sociodemographic and medical characteristics, their experiences with anonymity and attitudes towards anonymity. Due to the non-normal distribution of the data, median and ranges were calculated and non-parametric tests conducted. When no significant group differences were found, descriptive statistics for the whole sample are given, referred to as participants. For all analyses we used SPSS 25.0 (IBM) and a *p*-value less than 0.01 was considered statistically significant due to multiple testing.

## Results

In total, 354 donors and 204 recipients completed and returned the questionnaire (response rate 57 and 35% respectively). Socio-demographic and medical characteristics are presented in Table 1. Half of recipients had received their kidney through the UKLKSS while the other half had received a kidney from a NDAD *via* the UK transplant list. Amongst donors who completed the questionnaire, 63% were NDADs and 37% were paired-pooled donors. Donors were significantly older than recipients and significantly more likely to be male.

### Perceived Stress

Participants generally did not find the transplantation or donation stressful (Median = 2, IQR = 1–4). Recipients perceived the transplantation significantly more stressful (Median = 4, IQR = 2–5) than donors perception of donation (Median = 2, IQR = 1–3), *U* = 20,305, *p* = 0.000. No significant associations were found between perceived stress experiences with anonymity and attitude towards anonymity.

### Knowledge of the Official Policy on Anonymity

Forty-four percent of all participants falsely believed that anonymity was required before and after transplantation. The same proportion of participants (43%) correctly believed that anonymity was required before surgery, but that after surgery donor and recipient can meet if both parties agree. The remaining 13% did not know about an official policy on anonymity. Those who knew about the possibility to rescind anonymity were more likely to do so than those who did not know, χ^2^ = 17,231, *p* = 0.000.

### Experiences With Anonymity

The large majority of participants (among recipients 89%, among donors 92%) reported that anonymity was maintained. Looking back at the donation/transplantation, these donors (median = 7, IQR = 7–7) and recipients (median = 7, IQR = 6–7) were content with anonymity before surgery. The value of the mean ranks indicated that donors (Mean Rank = 256.4) were significantly more content with anonymity before surgery than recipients (Mean Rank = 227.2), *U* = 24,351, *p* = 0.002. These donors (median = 7, IQR = 5–7) and recipients (median = 7, IQR = 4–7) were also satisfied with anonymity after surgery. Time since surgery was not related to satisfaction with anonymity before or after surgery.

Of all participants who remained anonymous, only 6% would have liked to meet the other party before surgery and 37% would have liked to meet after surgery. This did not significantly differ between donors and recipients. However, if the other party would like to meet, openness to meeting them rises to 63%. This did not significantly differ between donors and recipients. No relationship was found between preferences for contact and time since surgery.

Half of all recipients who remained anonymous sent an anonymous card to their donor and 24% received an anonymous card from their donor. Amongst donors, 21% sent an anonymous card to their recipient and 34% received an anonymous message from the recipient. Recipients more often sent an anonymous card than donors did, χ^2^ = 44,453, *p* = 0.000.

### Experiences With Broken Anonymity Among Recipients

Amongst recipients, 22 (11%) reported that anonymity was broken, of which 9 only had contact with the donor in writing or on the phone, a median of 7 months after surgery (range 1–48). Twelve recipients actually met their donor, a median of 10 months after surgery (range 3–33). One recipient reported that he accidentally found out about his donor before surgery. It remains unclear how this has happened. These 22 recipients were generally very content with anonymity before surgery (Median = 7, IQR = 6–7) and with the broken anonymity after surgery (Median = 7, IQR = 7–7). All 22 recipients reported positive experiences with the contact/meeting they had with their donor (Median = 7, IQR = 7–7) and did not regret the contact/meeting (Median = 1, IQR = 1–1). Two recipients who came to know their donor after surgery, were discontent with anonymity before surgery. One of these recipients reported that she found the transplantation extremely stressful.

Major drivers of contact or meetings were the request of the recipient (n = 9) and his or her need to express their gratitude. Some recipients mention that the donor initiated contact (n = 3) or that both they and the donor initiated contact (n = 4). Three recipients accidentally found out who their donor was, due to inadvertent administrative errors or through talking with other patients on the ward. Quotations to illustrate recipients’ experiences with the contact or meeting they had with their donor can be found in Table 4.

**TABLE 4:** Quotations to illustrate recipients’ experiences with breaking anonymity.

Experiences with written correspondence only	
Male, NTL^1^	“I wanted to thank the donor and explain how they saved my life. We exchanged messages on social media and I sent a letter of thanks. I have chosen not to meet the donor (famous person) as TV would be involved and I would have no control over the TV editing.”
Female, PPD^2^	“I was curious to know who has been kind enough to donate a kidney to a stranger. I sent a thank you card, but was told there was a high possibility I wouldn’t hear back so when I received a very nice card and letter, I was extremely happy. The contact was only exchanging cards.”
Female, NTL	“I needed to express my gratitude and happiness. We exchanged letters. One letter each.”
Male, PPD	“My transplant co-ordinator sent me a letter that my donor wished to contact me by letter and email etc. We have never met, but we exchange Christmas cards and email.”
**Experiences with meeting in person**	
Female, PPD	“We were near each other on the ward and we got talking. It was not hard to work out.”
Female, NTL	“I wrote to say thank you and received lovely letters back. We met 18 months after and although we have very different lifestyles, we have the same values in life.”
Male, NTL	“I wanted to thank my donor for the fantastic gift of one of her kidneys. Because the whole process had been such a major-life event for both couples, we wanted to complete the experience by meeting at least once. I feel that it was beneficial to both parties to form some personal relationship to enhance the experience, I believe the donor would agree.”
Male, NTL	“Both myself and the donor wrote a letter to the transplant co-ordinator and after exchanging letters both parties wanted to meet each other. This was strictly connected through the hospital co-ordinator in case either party changed their minds prior to meeting each other.”
Female, PPD	“We met one of the couples at clinic, we recognized them as they had appeared on TV promoting transplant donations in the news. The meeting went well and we hugged and thanked each other. It was a three way transplant; have not met the other couple but cards and telephone connections have been exchanged by all three couples. It was a very positive experience. I was very pleased to make contact with both donation couples. Unfortunately, one recipient whose partner donated his kidney to me has since died of cancer and it was extremely upsetting to hear this bad news. It was good to exchange cards with all transplant couples, but to be aware that things don’t always go to plan.”
Male, NTL	“He changed my life and I wanted to show my gratitude. We met for lunch. My words were “I don’t know whether to shake your hand or hug you.” We shook hands, later we hugged in private. We have both undertaken skynews interviews (together). We bonded immediately but if meeting prior I would have felt under pressure. Suppose he didn’t like me!”
Female, PPD	“I wrote a card to send to my donor *via* the transplant nurse coordinator to say thank you. My donor was really happy I got in touch as she also wanted to find out more about me and eventually meet me.”

1 NTL, recipient on national transplant list.

2 PPD, recipient registered in paired/pooled donation.

### Experiences With Broken Anonymity Among Donors

Amongst donors, 29 (8%) reported that anonymity was broken after surgery, of which 17 only had contact with the recipient in writing or on the phone, a median of 9 months after surgery (range 1–30). Eleven donors actually met their recipient, a median of 12 months after surgery (range 5–36). One donor found out about his recipient through the newspaper (unclear if they had contact at all). In general, these donors were content with anonymity before surgery (Median = 7, IQR = 3–7). Most donors were very content about knowing their recipient afterwards (Median = 7, IQR = 7–7). Most donors experienced the contact/meeting with the recipient as very positive (Median = 7, IQR = 7–7) and did not regret the contact/meeting (Median = 1, IQR = 1–1). One donor felt neutral about the contact she had with her recipient (Median = 4) which was in the form of a formal written thank you card forwarded by the living donor coordinator. Two other donors reported to be only somewhat content with the fact that the recipient is no longer anonymous to them and regret the contact/meeting they had. In both cases, the recipient initiated contact with the donor. Nevertheless, for one of these donors contact with his recipient went beyond his expectations. A major motivation for donors to have contact with the recipient was their curiosity about the outcome. In most cases, the recipient initiated contact (n = 20), however some donors wanted to reassure themselves that the donation was successful and initiated contact with their recipients (n = 7). Quotations to illustrate donors’ experiences with the contact or meeting they had with their recipient can be found in Table 5.

**TABLE 5:** Quotations to illustrate donors’ experiences with breaking anonymity.

Experiences with written correspondence only	
Male, PPD^1^	“The recipient contacted me, but I was happy to hear from her to know that all was going well. It was rewarding to know of the benefits the transplant brought to the recipient and her family.”
Male, PPD	“The recipient contacted me through the transplant team. We have exchanged letter and e-mails. I don’t want to meet the recipient.”
Female, NDAD^2^	“I was keen to know the outcome for him or her (hopefully positive but wanted to know even if it’s not). We have exchanged emails and have spoken on the phone. I was thrilled to know how the donation has changed not just her life, but also that of her family. We exchange ‘anniversary’ emails, but may not ever meet.”
Male, NDAD	“He sent me a card *via* the hospital and a second card 1 year after donation. He wanted to thank me in person. I wrote to him my name, address and phone number. I told him a bit about myself. I thought that he would be interested. I never received a reply. I wrote another card at Christmas. I never received a reply. Maybe he does not like the Irish. For a little brief moment I felt snubbed, I’m only human.”
Female, PPD	“It was lovely to hear from the impact my gift made to the recipient, her immediate family and, particularly, to know she now hopes to see her grandchildren grow up. I’ve tried to keep in touch with my recipient, but all letters had to go between both coordinators. It felt stalled and eventually I broke contact.”
**Experiences with meeting in person**	
Female, PPD	“As we were part of a pairing scheme, we wanted to see how well they were doing and my husband wanted to thank his donor. We exchanged emails and met up approximately 10 months later.”
Male, NDAD	“I wanted to reassure myself that the operation was successful and to confine to myself that what I did was of some purpose. To see that the person was healthy now. They responded to my letter very favourably and wanted to meet me. We then visited each other’s families and have become friends.”
Female, PPD	“We had several contacts by card/letter *via* the transplant co-ordinators at the hospital. I offered my address to be forwarded to the recipient to make it easier and save NHS money. We had corresponded several times and found each other on Facebook before agreeing to meet. It is amazing to see now she has flourished since receiving the kidney.”
Female, NDAD	“I wanted to meet my recipient, because I was curious. She was such a genuine person; her gratitude made me feel good.”
Female, PPD	“I was part of a paired donation and my recipient’s wife contacted her recipient who gave me their details. I contacted them. My recipient’s wife suggested that we all meet up and it seemed like a good idea. We all met for a very emotional day. It was good to see my friend (we were not compatible) and my recipient looking so well.”
Male, NDAD	“I thought I preferred not to have contact as I did not wish to establish emotional ties. After exchange of correspondence, I agreed to break anonymity to meet, because it appeared very important to the recipient and his family. When this did happen, it was a very positive experience as the recipient and his family were delighted and clearly the transplant had been successful and their quality of life enhanced immeasurably.”
Male, NDAD	“My recipient wished for contact. The whole purpose of donation was to help someone so I wanted to give him the contact he wished for.”

1 PPD, donor registered in paired/pooled donation.

2 NDAD, non-directed altruistic donor.

### Attitudes Towards Anonymity

Fifty-eight percent of all participants were for anonymity between donors and unknown recipients, but 34% were not sure. A small group (8%) was against. There was no significant difference between donors and recipients. Table 6 shows that the distribution of opinions (for, against, not sure) among participants who broke anonymity was significantly different from those who remained anonymous: in the non-anonymous group, a higher number were against anonymity (21%) than in the anonymous group (6%, χ^2^ = 14,229, *p* = 0.001).

**TABLE 6:** General attitude towards anonymity among those who remained anonymous and those who broke anonymity.

Statement	Anonymity maintained *n* = 507		Anonymity broken *n* = 51	
I am for anonymity between living donors and unknown recipients	284	59%	22	46%
I am against anonymity between living donors and unknown recipients	29	6%	10	21%
I’m not sure	165	35%	16	33%
Missing	29		3	

The median attitudes towards anonymity are presented in Table 3. Participants agreed strongly with anonymity before the operation and believe that, if desired, a meeting should be allowed after surgery. Participants also agreed with the statement that anonymity makes a donation altruistic. There was less consensus on the statements that there must be anonymity after the surgery and whether surgery should only proceed if the donor agrees to anonymity. In general, participants did not agree that anonymity was difficult to maintain nor that removing anonymity would result in an increase in donors. Participants disagreed with donors and recipients having the right to know the other party.

We found no evidence for differences in attitudes between donors and recipients, except for one statement. Recipients agreed significantly more with the statement that “the donor has the right to know to whom he/she is donating a kidney” (Median = 2, IQR = 1–5) than donors (Median = 1, IQR = 1–3), *U* = 27,896, *p* = 0.000.

There were few differences in attitudes between participants who broke anonymity and participants who maintained anonymity. The anonymous group (Median = 4, IQR = 3–6) agreed significantly more with the statement that “there must be anonymity after the operation” than the non-anonymous group (Median = 2, IQR = 1–4), *U* = 6,761, *p* = 0.000. Non-anonymous participants (Median = 7, IQR = 6–7) also agreed more than anonymous participants (Median = 7, IQR = 4–7) with the statement that “if both parties agree, the donor and recipient should be allowed to meet after surgery,” *U* = 9596, *p* = 0.005. Likewise, non-anonymous participants (Median = 3, IQR = 1–5) agreed significantly more than participants who remained anonymous (Median = 1, IQR = 1–3) with the statement that in practice anonymity is difficult to maintain, *U* = 9,801, *p* = 0.005.

### Associations Between Attitudes and Socio-Demographic and Medical Characteristics

No significant relationships were found between attitudes toward anonymity and gender, education and time since donation. Participants’ age was significantly related to three statements. The older the participant, the more they agreed that there must be anonymity before the operation (*r*_s_ = 0.150, *p* = 0.001) and that the donation should only proceed if the donor agrees to anonymity (*r*_s_ = 0.145, *p* = 0.003). The younger the participant, the more they agreed that the donor has the right to know to whom he/she is donating a kidney (*r*_s_ = −0.131, *p* = 0.001).

We found no evidence that attitudes toward anonymity differed according to type of transplant program, except for the following two statements. NDADs (Med = 7, IQR = 6–7) agreed significantly more that there must be anonymity before the operation than paired-pooled donors (Med = 7, IQR = 4–7), *U* = 11,928, *p* = 0.009. Likewise, NDADs (Med = 5, IQR = 2–7) agreed significantly more that the donation should only proceed if the donor agrees to anonymity (Med = 3, IQR = 1–5), *U* = 10,314, *p* = 0.001.

## Discussion

This study shows that, despite the policy on anonymity in the UK, whereby anonymity can be broken after surgery with mutual consent, few donors and recipients make use of this possibility. Only 8% of donors and 11% of recipients reported that anonymity was broken in some way and only 3% of donors and 6% of recipient had met the other party. Experiences with broken anonymity were all positive and all donors and recipients who had contact with, or met the other party, had no regrets. Most participants reported that anonymity was maintained and that they were satisfied with the anonymity of their own procedure. In general, participants agreed strongly with anonymity before surgery. Opinions about whether there should be anonymity after surgery were mixed. However, all participants believed that a meeting should be allowed after surgery if both parties agree. This seems to indicate that participants are satisfied with the current conditional approach on anonymity.

It is remarkable that the opportunity to revoke anonymity is used so little. Another study among NDADs in the UK also found contact among donors and recipients to be minimal, with only 2% meeting in person (10). Partly, this might be due to participants having mistaken beliefs about the anonymity policy (44% of our participants falsely believed that anonymity was required before and after transplantation). As the possibility of breaking anonymity is discussed with all NDADs and donors and recipients participating in a KEP, it seems that this information is not retained or remembered by participants. It might also indicate that there is little demand for non-anonymous contact among participants in KEPs, because they are content with anonymity and/or because of anxiety about the consequences of breaking it (9).

A similar study has been conducted in the Netherlands and Sweden (where anonymity is perpetual), allowing a comparison of results (9). We found that experiences with anonymity and attitude towards anonymity are similar (9). Some exceptions include greater disagreement among UK participants that a meeting before surgery should be allowed if both parties agree. UK recipients also disagreed more with the statement that donors have the right to know to whom they are donating. However, in both studies, participants strongly believed that a meeting should be allowed after surgery, if both parties agree. This value of autonomous decision-making regarding maintaining or removing anonymity after surgery was also found in another prospective study on attitude towards anonymity among Dutch donors and recipients (8).

Another important finding is that, in all three studies we conducted on anonymity, most participants who remained anonymous did not want to meet their donor/recipient, but were more likely to accept a meeting if the other party would want to meet them (8, 9). This tendency to conform to the needs of the donor/recipient raises the question to what extent recipients and donors might be willing to shift (or even cross) their own boundaries and agree to have contact even when this is not their own personal desire. For recipients, this increased openness could result from a sense of indebtedness towards the donor (11), and for donors, this openness could be explained by their altruistic tendencies (12). However, our finding that written correspondence is more common than meeting directly suggests that donors and recipients do not ultimately all progress to meeting in person and could be interpreted as successful protection of personal boundaries.

One could argue that, since the initiation of KEPs, the discussion on the risks and benefits of anonymity in anonymous donation, has long been more speculative than evidence-based. Several papers described that revoking anonymity after surgery puts donors and recipients at risk for disappointment when the reality differs from an idealized image of recipient/donor or the outcome of the transplantation (7, 13, 14). Also that lifting anonymity could lead to a fall in donation rates, donors attempting to seek reward or to recipients feeling indebted to the donor, and that continued contact might obstruct both parties from achieving closure (7). Although only few studies report on actual experiences with broken anonymity, they provided little evidence for such negative experiences. Rodrigue et al. (15) reported positive experiences of altruistic donors in the US that made contact with their recipient after donation, and Maple et al. (10) found a high satisfaction with non-anonymity in the UK. We are unaware of any empirical studies on the impact of lifting anonymity on donation rates, but as long as maintaining anonymity remains an option, donation rates are unlikely to be influenced by a possible relinquishment of anonymity after donation (5, 7). This is supported by the rising number of unspecified donors in the UK (2, 5) and an increased interest in directed altruistic donation which allows living kidney donors to direct their altruistic donation to a specified recipient they did not know before (often presenting themselves online or in the media). Also, the current study does not support the aforementioned concerns on removal of anonymity in the context of a well-established living donation programme. Rather the findings support the current conditional approach to anonymity that leaves the option for voluntary contact and thereby tailors the donation/transplantation experience to the donors’ or recipients’ individual wishes to maintain or remove anonymity (if both parties agree). Nevertheless, in clinical practice we should support well-informed decision-making about (removal of) anonymity, by standardized approaches to removal of anonymity, education on risks and benefits of non-anonymous contact between pairs, and pre-operative counselling for donors and recipients.

Despite the strengths of our study, being the first survey study to investigate the issue of anonymity among participants of NDAD and the UKLKSS in the UK, and a large sample size, some limitations should be taken into consideration. Firstly, we did not perform a formal validity assessment of the questionnaire, however using the same questionnaire as previous studies allowed comparison of results. Secondly, the retrospective nature of some questions might have introduced recall bias, as for some participants surgery was almost 8 years ago. However, we found no associations between time since survey and any outcome measures. Thirdly, the response rate was not as high as we hoped for, especially among recipients. This might have introduced a nonresponse bias, for example among those with less positive experiences, and might limit the generalizability of our findings. Another possible limitation of the study is related to the definition of anonymity which may vary between professionals and donors and recipients. Mamode et al. (7) wrote that “anonymity in the context of transplantation could be considered as a situation in which personally identifiable information about the donor is not known by the recipient and vice versa.” Based on the responses it appeared that some participants interpreted anonymity differently than we had intended. In this study we chose to follow the perception of the participants and grouped them accordingly in the anonymous or non-anonymous group.

In conclusion, this study demonstrated high satisfaction among UK donors and recipients with the current conditional policy on anonymity, leaving the option for voluntary contact post-transplant. There was little contact between donors and recipients, but the reported experiences with breaking anonymity were positive. These and earlier findings support conditional anonymity over absolute anonymity and are informative for other countries who are assessing current policy on anonymity or considering developing an anonymous living donation programme. Clearly, standardized approaches to removal of anonymity after surgery, education on the advantages and disadvantages of having contact for patients and donors as well as guidelines for healthcare professionals are of utmost importance.

## Capsule Summary Sentence

Currently, almost 5,000 people in the UK are waiting for a kidney transplant. With the national living kidney sharing scheme it is possible to share donated kidneys across the UK. This increases the number of transplantations than can be carried out. The scheme relies upon anonymity between donor and recipient pairs to avoid disclosure of identity before transplantation. Anonymity has long been seen as a way to prevent commercialization of organs, to prevent disappointment in case of poor recipient outcome, and to protect donor/recipient identity. Unlike in other European countries, in the UK anonymity can be broken with the consent of all parties after the transplantation. As one of few studies, we provide evidence on how often anonymity is broken in the UK and how broken/maintained anonymity is experienced by participants in the kidney sharing scheme. We previously conducted the same study in the Netherlands and Sweden, where it is not allowed to break anonymity after transplantation. Based on these two studies, we make the case that the option of breaking anonymity after transplantation can safely be adopted by other countries in which kidney sharing schemes exist or are to be developed.

## Data Availability

The raw data supporting the conclusions of this article will be made available by the authors, without undue reservation.

## Abbreviations

ELPAT, Ethical, Legal and Psychosocial Aspects of Organ Transplantation; KEP, kidney exchange programme; LDKT, living donor kidney transplantation; NDAD, non-directed altruistic donation or donor; UKD, unspecified kidney donation or donor; UKLKSS, UK living kidney sharing scheme.
